# Account of a Profitable Method of Obtaining Phosphoric Acid

**Published:** 1800-11

**Authors:** 


### *Account of a profitable Method of obtaining Phosphoric Acid. By* Mr. Suersen.

[Extracted from the Northern Archives of Medicine, &c. in German, Vol. I. No. 2.]

THE shortest way of preparing pure phosphoric acid, is, undoubtedly, by acidifying the phosphorus with nitric acid, which is done with the least loss of time, and without great expence. Although this process has been generally thought to require the utmost caution, it is only necessary when a great portion of phosphorus is infused with nitric acid; as by the vehement action of such a quantity of phosphorus upon the nitric acid, a part of it rushes out, and by getting in contact with atmospheric air, catches fire, and blows up the vessel in which the distillation is performed. The same accident happens when, according to Hermbstaedt's experiment, four ounces of nitrous acid, diluted with eighteen ounces of water, are poured upon one ounce of phosphorus, and distilled in the sand bath. There is, at first, no perceptible activity upon the acid; but when this becomes by degrees more concentrated, a similar effect, as in the former operation, is produced. The process, as proposed by M. Suersen, is the following: Several ounces of pure concentrated nitric acid, after being diluted with equal parts of water, are so divided, that each single ounce is poured into a separate subliming glass, and after having put them upon an iron grate, half a drachm of phoſphorus is added to each portion of the nitric acid, and the veſſels are heated by means of a lamp, till a ſufficient action of the phoſphorus on the nitric acid is perceived, by diſcharging a conſiderable quantity of nitrous fumes, but without becoming too vehement; and, in caſe the fumes ſhould begin to illuminate, any accident that accompanies the former methods may be prevented by pouring a little diſtilled water on the mixture, and, at all events, the lamp may be laid aſide when the veſſels ſeem to be ſufficiently heated. The addition of the phoſphorus is now continued in ſmall portions, and the veſſels alternately heated, till a ſufficient quantity of it is acidified. For the acidification of a conſiderable portion of phoſphorus, four ounces of nitrous acid are generally required of 1,508 ſpecific gravity, at a temperature of 61° Fahrenheit, and it may be completed in the ſpace of four hours. The phoſphoric acid thus obtained is very diluted, and muſt be expoſed to a diſtillation in the ſand bath; while it continues there a phoſphoric acid is obtained, of the conſiſtency of a ſyrup, whoſe ſpecific weight is about 2000. By this method two ounces and a half of a very concentrated and pure phoſphoric acid are obtained from one ounce of phoſphorus.

CRITICAL

